# Potential role of “the compensating benefit” in claiming non-inferiority

**DOI:** 10.1186/1745-6215-14-S1-P12

**Published:** 2013-11-29

**Authors:** Primrose Beryl, Werner Vach

**Affiliations:** 1 Institute of Medical Biometry and Medical Informatics, University Medical Center, Freiburg, Germany

## 

Non-inferiority (NI) trials attempt to demonstrate that the loss in efficacy by a new treatment compared to a standard treatment is limited to a certain pre-specified margin, usually compensated by some form of benefit in the form of safety, ease in administration, tolerability, costs, etc. The lack of mention of the benefit of the new treatment and its importance have been pointed out recently.

The concept of ‘relative efficacy’ is highly relevant in non-inferiority trials as the trade-off between the benefit gained and the loss in efficacy plays an important part in the non-inferiority claim. Applying the concept of incremental cost effectiveness ratio, used widely in health economics, in a non-inferiority setup, we propose the benefit efficacy ratio as the ratio of gain in benefit to loss in efficacy for the new treatment versus the standard treatment. The ratio allows the benefit to contribute to the statistical claim of non-inferiority and the patient / physician to judge for oneself. We have studied the feasibility of this concept among a set of NI trials published in four major medical journals from 2005 to 2011.

Among 112 published NI trials, potential benefits could be perceived in 89(80%) trials of which, data related to any of the benefit was available in 56(63%) trial reports. Both efficacy and benefit favoured the new treatment among 29(54%) trials and both favoured the control treatment in 4(7%). By its application in these trials, we show that the benefit efficacy ratio could help overcome certain glitches of non-inferiority.

